# An outlook on clinical and demographic variables in patients with severe COVID-19 across different waves in Mexico and SARS-CoV-2 variants: data from two high-specialty centers

**DOI:** 10.3389/fpubh.2026.1794262

**Published:** 2026-04-23

**Authors:** Ingrid Fricke-Galindo, Ilse Adriana Gutiérrez-Pérez, Diana Lizbeth Ortíz-Farias, Esperanza Figueroa-Hurtado, Iris Paola Guzmán-Guzmán, Gloria Pérez-Rubio, Ivette Buendía-Roldán, Leslie Chavez-Galan, Arturo Cortés-Telles, Ramcés Falfán-Valencia

**Affiliations:** 1 Pneumogenomics Laboratory, Instituto Nacional de Enfermedades Respiratorias Ismael Cosío Villegas, Mexico City, Mexico; 2 Faculty of Chemical-Biological Sciences, Universidad Autónoma de Guerrero, Chilpancingo, Guerrero, Mexico; 3 Clínica de Enfermedades Respiratorias, Hospital Regional de alta Especialidad de la Península de Yucatán–IMSS Bienestar, Mérida, Yucatán, Mexico; 4 Translational Research Laboratory on Aging and Pulmonary Fibrosis, Instituto Nacional de Enfermedades Respiratorias Ismael Cosío Villegas, Mexico City, Mexico; 5 Laboratory of Integrative Immunology, Instituto Nacional de Enfermedades Respiratorias Ismael Cosío Villegas, Mexico City, Mexico

**Keywords:** clinical variables, COVID-19, pandemic, SARS-CoV-2, vaccine

## Abstract

**Background:**

Throughout the COVID-19 pandemic, there was variability in outcomes and in the clinical and demographic characteristics of patients with severe disease. We aimed to compare clinical and demographic variables at hospital admission among patients with severe COVID-19 at two high-specialty centers in Mexico during the pandemic waves, accounting for relevant virus variants and patients' vaccination status.

**Methods:**

This retrospective, descriptive study included medical records of 1,425 patients hospitalized with COVID-19 (March 2020–November 2022) at the National Institute of Respiratory Diseases (INER, Mexico City, Mexico) and the Regional High Specialty Hospital of the Yucatán Peninsula–IMSS Bienestar (Merida, Yucatán, Mexico). The comparison was performed between the 1st wave, the 2nd and 3rd waves, and the 4th to 6th waves in Mexico, among the pre-dominant SARS-CoV-2 variants (Delta and Omicron), and vaccination status during the Omicron stage.

**Results:**

At the 1st wave, patients were pre-dominantly male and younger; whereas in the 4th to 6th waves, a high frequency of subjects with heart disease was observed. The longest hospital stay (median 23 days) and the highest percentage of invasive mechanical ventilation (81.02%) were observed during the 2nd and 3rd waves. Heart diseases and D-dimer were relevant during the Omicron stage. Clinical and demographic variables were similar between unvaccinated and vaccinated subjects, although the mortality was lower in the vaccinated group.

**Conclusions:**

These differences in the studied variables across pandemic waves and SARS-CoV-2 variants help explain the variability in clinical outcomes throughout the COVID-19 pandemic.

## 1 Introduction

The public health emergency due to coronavirus disease (COVID-19) lasted 3 years and 3 months, and the pandemic was among the deadliest infectious diseases in recent history (1). As of 2025, more than 778 million COVID-19 cases have been confirmed, with more than 7 million deaths, and 13.64 billion COVID-19 vaccine doses administered (2).

The etiological agent was named severe acute respiratory syndrome coronavirus (SARS-CoV-2), which belongs to the Betacoronavirus genus and the Coronaviridae family (3). The virus particles are spherical or pleomorphic, with a diameter of about 60–140 nm. It has one of the largest single-strand RNA genomes with 27–32 kilobases, encoding four major structural proteins: spike protein (S), membrane protein (M), envelope protein (E), and nucleocapsid protein (*N*) (4).

The SARS-CoV-2 virus quickly spreads worldwide. Its efficient viral structure enabled it to bind effectively to the ACE2 receptor on human cells, with the help of host cell enzymes such as TMPRSS2 and furin. It was also highly contagious from person to person through respiratory droplets and aerosols. The virus demonstrated significant environmental stability, as it can survive for varying periods on different surfaces (5).

By the end of 2020, SARS-CoV-2 had adapted better to humans after circulating throughout the year. Various highly mutated forms of the virus evolved, increasing their transmissibility, and were labeled as “variants of concern” (VOCs). Therefore, Alpha (B.1.1.7), Gamma (P.1), Delta (B.1.617.2), and Omicron BA.1 (B.1.1.529) and BA.2 (B.1.1.529.2) viruses were the VOCs that represented the COVID-19 pandemic (6). A study in a golden hamster model evaluated the pathogenicity and potential respiratory transmission of the circulating variants. The authors found that Delta, BA.1, and BA.2 replicated more efficiently than the other variants; while Gamma and Delta were transmitted more efficiently and caused greater lung pathology, BA.1 and BA.2 were less efficiently transmitted via the respiratory system (7).

Variability in the virus's pathogenicity and transmissibility also affected the clinical characteristics of patients with COVID-19 across different waves. The COVID-19 wave was marked by a clear rise in cases, with variation across the globe (8). In Mexico, the first case of COVID-19 was confirmed on February 28th, 2020 (9), and six pandemic waves were registered in the country until the declaration of the end of COVID-19 as a public health emergency on May 5th, 2023 (10).

Several clinical and demographic variables were associated with mortality rate in Mexico, such as being ≥50 years, obesity, diabetes, hypertension, and chronic kidney disease (11); and a study showed that the combination of these risk factors increased the fatality rate (12). Likewise, several laboratory parameters and systemic inflammation biomarkers were identified as risk and prognostic factors of COVID-19 outcome. Thus, leukocytosis, lymphocytopenia, elevated D-dimer, C-reactive protein (CRP), lactate dehydrogenase (LDH), and ferritin were associated with disease severity and progression (13, 14), as well as indices such as neutrophil-to-hemoglobin and lymphocyte (NHL), neutrophil to lymphocyte ratio (NLR), and red blood cell distribution width (RDW) were observed as predictive factors of COVID-19 severity and mortality (15).

The SARS-CoV-2 variants exhibited different propagation rates, associated disease severity, performance of testing tools, and treatment response (16, 17). Likewise, the pandemic waves exhibited differences in COVID-19 transmissibility and severity due to social and public health responses (17); however, the clinical variability among patients hospitalized during different COVID-19 stages at the end of the pandemic has not been reported. We aimed to compare clinical and demographic variables at hospital admission among patients with severe COVID-19 across two third-level reference centers in Mexico during the pandemic waves and relevant VOCs, and by vaccination status.

Mexico faces great challenges in access to health care and presents high rates of crowding conditions and poverty, which severely affect the clinical outcome of patients with COVID-19 hospitalized during the pandemic (18). The two high-specialty centers included in this study are in Mexico City and Yucatán, Mexico. The former is located at the center of the country, is the premier institution for respiratory diseases in Mexico, and was treating the most severe cases of COVID-19 during the pandemic, with more than 200 beds available for critical procedures such as invasive mechanical ventilation (19). Meanwhile, the high-specialty center from Yucatan is located in southeastern Mexico, a region characterized by an aging population, a high prevalence of comorbidities, and significant socioeconomic disparities in rural and indigenous areas (20).

## 2 Methods

### 2.1 Study design

This is a retrospective, descriptive study of medical records documentation of patients hospitalized with severe COVID-19 (from March 2020 to November 2022) at two third-level reference centers in Mexico: the National Institute of Respiratory Diseases (Instituto Nacional de Enfermedades Respiratorias Ismael Cosio Villegas, “INER”, Mexico City; Mexico) and the Regional High Specialty Hospital of the Yucatán Peninsula–IMSS Bienestar (Hospital Regional de Alta Especialidad de la Península de Yucatán, “HRAEPY”, Mérida, Yucatán; México). The study was approved by the Local Ethics Committees (No. C53–20 and B11–23) and conducted in accordance with the Helsinki Declaration (21).

### 2.2 Study settings

A total of 1,463 electronic records were consulted. We included only patients >18 years old with a confirmed diagnosis of COVID-19 by RT-PCR SARS-CoV-2 test and for whom clinical laboratory data were available in electronic records. According to the inclusion criteria, 38 records (2.5%) were discarded. Thus, 1,425 electronic records were considered for the study. The data collected for each patient were: age, sex, co-morbidities (type 2 diabetes mellitus, obesity, chronic respiratory diseases, heart disease, and systemic arterial hypertension), clinical information from patients at hospital admission (oxygenation data, white blood cell count, C-reactive protein, and D-dimer levels), and the clinical outcomes (mortality, requirement of invasive mechanical ventilation, and length of hospital stay in days). Then, they were classified according to the following criteria (Table 1, Supplementary Figure 1): (1) pandemic waves in Mexico grouped by their epidemiological relevance (10); (2) pre-dominant SARS-CoV-2 variants (22) during the hospital admission of the patient; and (3) vaccination status after the COVID-19 vaccine was available (23).

**Table 1:** Classification criteria of patients' data included in the study.

Pandemic waves in Mexico			
	1st wave	2nd and 3rd waves	4th to 6th waves
Start and end dates of the waves^a^	02/16/2020–09/26/2020	09/27/2020–04/17/2021 and 06/06/2021–10/23/2021	12/19/2021–03/05/2022, 05/29/2022–08/20/2022, and 12/04/2022–01/28/2023
**Pre-dominant SARS-CoV-2 variants^b^**			
	**Other^c^**	**Delta**	**Omicron**
Frequency	Other variants 72.4–99.4%	Delta variant 20.4–97.4%	Omicron variant 61.0–100.0%
Date covered	03/28/2020–04/27/2021	06/04/2021–09/20/2021	12/30/2021–11/15/2022
**Vaccination status after the COVID-19 vaccine was available^d^**			
	Unvaccinated	Vaccinated	
Vaccination started 12/24/2020	Patients who did not receive any dose of the COVID-19 vaccine	Patients who received at least one dose of any of the COVID-19 vaccines	

^a^Dates of the beginning and end of each wave extracted from national documentation (10).

^b^Pre-dominant variant reported at the date of the patient's hospital admission, according to national documentation (19).

^c^SARS-CoV-2 variants not classified as a variant of concern (VOC), variant of interest (VOI), nor variant under monitoring (VUM) by the World Health Organization.

^d^In Mexico, the first COVID-19 vaccine dose was applied on December 24th, 2020.

The first vaccination campaign was for the healthcare professionals.

### 2.3 Statistical analysis

Data collection was automated and summarized in a database through the electronic medical record system. The categorical data are presented as absolute numbers and percentages. The median and interquartile range (Q1–Q3) are used for continuous data. The Kolmogorov-Smirnov test was used to assess normality. As applicable, Fisher's Exact, Mann-Whitney U, and/or Kruskal-Wallis tests were used for the comparison of the data. We performed a generalized linear model to adjust for the high-specialty center from which the data were obtained, and the specific group in each case (waves, virus variant, vaccination status) was included as a predictor variable. The handling of missing data used the listwise exclusion technique; thus, analyses were performed only for cases with complete data for the required variables. A significant *p*-value was set at *p* < 0.05. The statistical analyses were performed in RStudio v. 4.4.0 (24).

## 3 Results

### 3.1 Clinical and demographic characteristics of the patients with COVID-19 from the two high-speciality centres included in the study

Table 2 shows the clinical and demographic characteristics of patients with COVID-19, classified by the attending high-specialty centre. Patients from the HRAEPY were slightly younger than the patients hospitalized in the INER. The frequency of heart disease was the only comorbidity that differed between the two centers, being more common in Yucatán. Meanwhile, the clinical outcomes differed between the centers: cases of severe COVID-19 were more frequent in INER (longer hospital stays and more frequent IMV) than in HRAEPY, but mortality was higher in the center from Yucatan.

**Table 2:** Clinical and demographic variables among patients with severe COVID-19, classified by the attending high-specialty center.

Variable	HRAEPY (*n* = 368)	INER (*n* = 1,057)	*p*-value
Age, years	56 [44–68]	59 [50–69]	**<0.001**
Sex, males *n* (%)	234 (63.59)	689 (65.18)	0.612
T2D, *n* (%)	113 (30.71)	316 (29.90)	0.792
Obesity, *n* (%)	172 (46.74)	454 (42.95)	0.223
CRD, *n* (%)	16 (4.35)	73 (6.91)	0.103
HD, *n* (%)	32 (8.70)	42 (3.97)	**0.001**
SAH, *n* (%)	130 (35.33)	388 (36.71)	0.66
Hosp_stay, days	5 [3–11]	23 [16–37]	**<0.001**
Mortality, *n* (%)	151 (41.03)	368 (34.81)	**0.038**
IMV, *n* (%)	139 (37.77)	866 (81.93)	**<0.001**
Leukocyte (cell/μl)	10,955 [7,905–14,850]	10,000 [7,680–12,920]	**0.001**
Neutrophil (cell/μl)	8,845 [5,935–12,872]	8,500 [6,300–11,500]	0.108
Lymphocyte (cell/μl)	950 [647–1,470]	700 [400–1,000]	**<0.001**
Eosinophils (cell/μl)	10 [0–70]	0 [0–40]	**<0.001**
NLR	9.50 [4.97–16.75]	12.75 [7.42–22.47]	**<0.001**
Platelets (cell/μl)	266,000 [198,000–355,000]	268,000 [201,000–340,450]	0.585
CRP (mg/L)	137 [66–251]	104 [42–179]	**<0.001**
LCR	6.95 [3.17–19.46]	6.31 [3.18–19.23]	0.628
D-dimer (ng/ml)	1,099 [460–2,540]	1,330 [590–3,150]	**0.004**

Quantitative data are presented as median (interquartile range Q1–Q3), and qualitative data as absolute numbers (frequency in percentage).

The comparisons were performed using the chi-squared and Mann–Whitney U-tests. *p*-values <0.05 are indicated in bold style.

CRD, chronic respiratory diseases; CRP, C-reactive protein; HD, heart diseases; Hosp_stay, days of hospital stay; HRAEPY, Regional High Specialty Hospital of the Yucatán Peninsula–IMSS Bienestar; INER, National Institute of Respiratory Diseases, IMV, invasive mechanical ventilation; INER, LCR, lymphocyte-to-C-reactive protein ratio; NLR, neutrophil-to-lymphocyte ratio; SAH, systemic arterial hypertension; T2DM, type 2 diabetes mellitus.

In addition, some differences were observed for the laboratory parameters at clinical admission. The leukocyte, lymphocyte, and eosinophil counts were higher in HRAPEY patients than in patients from the INER, as were the C-reactive protein (CRP) values. Meanwhile, platelet and D-dimer parameters were higher in the INER than in the HRAEPY patients. Because clinical and demographic variables differed between the two centers, we adjusted for these covariates in the subsequent analyses.

### 3.2 At the 1st wave, patients were younger and pre-dominantly males when compared to the other waves, and a high frequency of heart diseases characterized the 4th to 6th waves

Distinct epidemiological waves characterized the timeline of the COVID-19 pandemic, and the clinical characteristics of patients with severe disease varied. Since data were obtained from hospitalized patients with COVID-19, the results reflect a population with severe disease.

In the first wave, patients were younger than those in the other groups, but age was not significant when comparing the 2nd and 3rd waves vs. the 4th to 6th waves. Meanwhile, the proportion of males decreased significantly in the last waves (4th to 6th) compared with the first three waves (Table 3).

**Table 3:** Comparison of clinical and demographic variables among patients with severe COVID-19, stratified by pandemic wave.

Variable	1st wave (*n* = 201)	2nd and 3rd waves (*n* = 1,038)	4th to 6th waves (*n* = 118)	*p*-value	Adjusted *p*-value
Age, years	54 (41–65)	60 (50–69)	63 (45–72)	**<0.001**^a^^,^ ^b^	**0.002** ^a^
Sex, males *n* (%)	140 (69.65)	679 (65.41)	64 (54.24)	**0.018**^b^^,^ ^c^	**0.006** ^b^
T2D, *n* (%)	52 (25.87)	318 (30.63)	37 (31.35)	0.377	**0.003** ^b^
Obesity, *n* (%)	105 (52.24)	453 (43.64)	43 (36.44)	**0.016**^a^^,^ ^b^	NS
CRD, *n* (%)	5 (2.49)	70 (6.74)	9 (7.63)	0.058	**0.040** ^b^
HD, *n* (%)	5 (2.49)	49 (4.72)	19 (16.10)	**<0.001**^b^^,^ ^c^	**<0.001**^b^^,^ ^c^
SAH, *n* (%)	60 (29.85)	392 (37.76)	47 (39.83)	0.08	**0.033** ^a^
Hosp_stay, days	7 (4–12)	23 (15–36)	4 (2–7)	**<0.001** ^a−*c*^	NS
Mortality, *n* (%)	77 (38.31)	381 (36.70)	49 (41.52)	0.565	NS
IMV, *n* (%)	87 (43.28)	841 (81.02)	29 (24.58)	**<0.001** ^a−*c*^	**0.001**^b^^,^ ^c^
Leukocyte (cell/μl)	10,790 (7,870–14,240)	10,000 (7,700–13,175)	10,990 (7,875–15,030)	0.08	NS
Neutrophil (cell/μl)	8,710 (5,560–12,230)	8,600 (6,208–11,675)	8,785 (6,050–13,268)	0.591	NS
Lymphocyte (cell/μl)	1,040 (740–1,570)	700 (400–1,000)	875 (542–1,372)	**<0.001** ^a−*c*^	NS
Eosinophils (cell/μl)	10 (0–40)	0 (0–57)	10 (0–90)	**<0.001**^a^^,^ ^c^	NS
NLR	7.91 (4.26–14.13)	12.75 (7.33–22.00)	11.21 (5.91–19.98)	**<0.001**^a^^,^ ^b^	**0.003** ^b^
Platelets (cell/μl)	280,000 (219,000–358,000)	266,000 (200,000–340,900)	252,000 (160,500–339,250)	**0.005**^a^^,^ ^b^	**<0.001** ^b^
CRP (mg/L)	150 (75–263)	104.6 (41.80–182.40)	108.2 (53.7–216.3)	**<0.001**^a^^,^ ^b^	NS
LCR	7.10 (3.42–17.16)	6.48 (3.18–19.97)	6.96 (3.05–23.23)	0.931	NS
D-dimer (ng/ml)	600 (330–1,762)	1,350 (590–3,070)	1,916 (1,039–3,870)	**<0.001** ^a−*c*^	**<0.001** ^b^

Quantitative data are presented as median (interquartile range Q1–Q3), and qualitative data as absolute numbers (frequency in percentage).

The comparisons were performed using the chi-squared and Kruskal–Wallis tests, with Bonferroni's *post hoc* test.

A generalized linear model was performed to adjust for the high-specialty center.

The *p*-value shows the comparison among the three groups; the superscript numbers indicate which groups differ. *p*-values <0.05 are indicated in bold style.

^a^First wave vs. 2nd and 3rd waves.

^b^First wave vs. 4th to 6th waves.

^c^Second and 3rd waves vs. 4th to 6th waves.

CRD, chronic respiratory diseases; CRP, C-reactive protein; HD, heart diseases; Hosp_stay, days of hospital stay; IMV, invasive mechanical ventilation; LCR, lymphocyte-to-C-reactive protein ratio; NLR, neutrophil-to-lymphocyte ratio; NS, not significant; SAH, systemic arterial hypertension; T2DM, type 2 diabetes mellitus.

Obesity was distributed differently across the grouped waves, but this difference was not observed after adjusting for the high-specialty center. The frequencies of the comorbidities type 2 diabetes mellitus (T2DM), chronic obstructive pulmonary disease (COPD), and systemic arterial hypertension (SAH) differed among the wave groups after adjustment for high-specialty center. Particularly, heart disease was more frequent in the last waves (4th to 6th) than in the 1st, 2nd, and 3rd.

The clinical outcomes differed across the grouped waves. The worst scenario was observed for the 2nd and 3rd waves, with the highest percentage of patients requiring invasive mechanical ventilation (81.02%). Nevertheless, the mortality remained constant throughout the pandemic waves (Table 3).

Regarding clinical parameters at hospital admission, leukocyte and neutrophil counts were not different across waves; likewise, lymphocyte and eosinophil counts were not different after adjusting for high-specialty center. A trend toward lymphopenia was observed across COVID-19 waves, with the lowest levels occurring after the 1st wave. Although platelet levels were normal, they were higher in the 1st wave and then gradually decreased.

The evaluated inflammatory biomarkers across the different waves revealed differential inflammatory processes during the COVID-19 waves. The NLR was overall high but notably increased after the 1st wave, and the difference remained after adjusting for the high-specialty center. By contrast, the highest levels of CRP were observed at the start of the pandemic, when compared to the remaining waves. Meanwhile, the lymphocyte-to-C-reactive protein ratio (LCR) did not differ among the grouped waves.

Increased D-dimer levels indicated activation of the hemostatic system during the pandemic, with levels becoming more pronounced as the pandemic progressed, with the highest levels observed in patients tested during the 4th to 6th waves (Table 3).

### 3.3 Older age, mortality, and D-dimer were relevant in the severe COVID-19 cases when Omicron was the pre-dominant variant during the pandemic

When patients were classified by pre-dominant SARS-CoV-2 variant, no significant differences in age were observed; however, patients were older during the Omicron stage when we adjusted for the high specialty center. Sex and the studied comorbidities were not relevant at any stage of the variant. However, the frequency of heart disease was notably higher (16%) in the Omicron era than in the Delta and other variants (4.95 and 1.46%, respectively) at the early stages of the pandemic. As disease severity indicators, the longest hospital stay was observed among patients hospitalized during Delta-variant pre-dominance (median of 29 days), as was the requirement for invasive mechanical ventilation (82.93%). However, the highest mortality was reported when the Omicron variant was pre-dominant (41.52 vs. 38.38% and 26.89% for other and Delta variants, respectively) (Table 4).

**Table 4:** Comparison of clinical and demographic variables among patients with severe COVID-19, classified by prevalent SARS-CoV-2 variants.

Variable	Other variants (*n* = 1,030)	Delta (*n* = 205)	Omicron (*n* = 118)	*p*-value	Adjusted *p*-value
Age, years	59 (50–68)	57 (45–71)	63 (45–72)	0.470	**0.005** ^c^
Sex, males *n* (%)	689 (66.89)	127 (61.95)	64 (54.24)	**0.015** ^b^	NS
T2DM, *n* (%)	296 (28.74)	73 (35.61)	37 (31.35)	0.138	NS
Obesity, *n* (%)	462 (44.85)	95 (46.34)	43 (36.44)	0.180	NS
CRD, *n* (%)	62 (6.02)	13 (6.34)	9 (7.63)	0.793	NS
HD, *n* (%)	51 (4.95)	3 (1.46)	19 (16.10)	**<0.001** ^a−*c*^	NS
SAH, *n* (%)	373 (36.21)	77 (37.56)	47 (39.83)	0.716	NS
Hosp_stay, days	19 (11–30)	29 (18–50)	4 (2.25–7.00)	**<0.001** ^a−*c*^	**<0.001** ^a−*c*^
Mortality, *n* (%)	400 (38.83)	57 (27.80)	49 (41.52)	**0.007**^a^^,^ ^c^	**<0.05**^a^^,^ ^c^
IMV, *n* (%)	755 (73.30)	170 (82.93)	29 (24.58)	**<0.001** ^a−*c*^	**0.008** ^c^
Leukocyte (cell/μl)	10,290 (7,800–13,700)	9,680 (6,830–11,960)	10,990 (7,875–15,030)	**0.002**^a^^,^ ^c^	**0.043** ^a^
Neutrophil (cell/μl)	8,800 (6,340–11,900)	7,800 (5,200–10,600)	8,785 (6,050–13,268)	**0.004**^a^^,^ ^c^	**<0.05**^a^^,^ ^c^
Lymphocyte (cell/μl)	700 (500–1,100)	700 (400–1,100)	875 (542.5–1,372.5)	0.075	NS
Eosinophils (cell/μl)	0.0 (0.0–40)	0.0 (0.0–100)	10 (0.0–90)	**<0.001**^b^^,^ ^c^	NS
NLR	12.11 (6.86–20.75)	10.15 (6.04–19.40)	11.21 (5.91–19.98)	0.124	NS
Platelets (cell/μl)	266,000 (201,000–338,000)	276,300 (207,100–358,900)	252,000 (160,500–339,250)	**0.044**^b^^,^ ^c^	**<0.001** ^c^
CRP (mg/L)	119.10 (59.75–210.05)	35.70 (13.11–79.05)	108.2 (53.7–216.3)	**<0.001**^a^^,^ ^c^	**<0.001** ^a^
LCR	5.98 (3.04–15.55)	22.51 (6.06–53.82)	6.96 (3.05–23.23)	**<0.001**^a^^,^ ^c^	NS
D-dimer (ng/ml)	1,260 (530–2,900)	910 (450–2,425)	1,916 (1,039–3,870)	**<0.002**^b^^,^ ^c^	**0.031** ^c^

Quantitative data are presented as median (interquartile range Q1–Q3), and qualitative data as absolute numbers (frequency in percentage).

The comparisons were performed using the chi-square and Kruskal–Wallis tests, with Bonferroni's *post hoc* test.

A generalized linear model was performed to adjust for the high-specialty center.

The *p*-value shows the comparison among the three groups; the superscript numbers indicate which groups differ according to. *p*-values <0.05 are indicated in bold style.

^a^Other variants vs. Delta variant.

^b^Other variants vs. Omicron variant.

^c^Delta variant vs. Omicron variant.

CRD, chronic respiratory diseases; CRP, C-reactive protein; HD, heart diseases; Hosp_stay, days of hospital stay; IMV, invasive mechanical ventilation; LCR, lymphocyte-to-C-reactive protein ratio; NLR, neutrophil-to-lymphocyte ratio; NS, not significant; SAH, systemic arterial hypertension; T2DM, type 2 diabetes mellitus.

Cell count values also vary by the pre-dominant SARS-CoV-2 variant group. The lowest leukocyte and neutrophil counts were observed during the Delta stage, although neutrophilia (>7,000 cells/μl) was present in all cases. Lymphopenia and elevated NLR were observed in all comparison groups. Meanwhile, eosinophil and platelet counts were within the normal range, but the values in patients hospitalized during the Omicron period differed from those in the other two variant groups. The inflammatory process was evident in all groups, with lower CRP levels and the highest LCR during the infection stage with the Delta variant. Finally, D-dimer levels were also particularly high during the infection stage with the Omicron variant (Table 4).

### 3.4 Clinical and demographic variables were similar between unvaccinated and vaccinated subjects with COVID-19 hospitalized during the Delta and Omicron stages

In Mexico, the first anti-SARS-CoV-2 vaccine was administered on December 24th, 2020. The initial vaccination campaign was aimed at healthcare professionals. In March 2021, the strategy was to start vaccinating the population aged 60 and older, followed by the rest of the population by decade, in descending order (25). According to this, the vaccination campaign was ongoing as the Delta variant evolved. At this stage, 36.10% (*n* = 74) of the patients herein studied, classified as Delta, had received at least one dose of the COVID-19 vaccine. Among these patients, 66.22% received one dose, 32.43% received two doses, and only 1.35% received three doses. Patients received different vaccines, including those from Oxford-AstraZeneca, CanSino Biologics, Gamaleya Center, and Pfizer–BioNTech.

The clinical and demographic variables for the groups stratified by vaccination status are shown in Table 5. During the Delta stage, vaccinated patients were older and had a higher frequency of T2DM and SAH than those who had not received a vaccine and were hospitalized due to COVID-19. This mainly reflects the strategy of vaccinating older people and/or those with comorbidities, who were identified as the more vulnerable population for COVID-19 severity and mortality. Nevertheless, no differences were observed in the groups' clinical outcomes. The in-hospital mortality rate and the need for invasive mechanical ventilation were similar between vaccinated and unvaccinated patients. Moreover, the longest hospital stay was observed during this stage (Table 3), but this was not affected by vaccination status.

**Table 5:** Comparison of clinical and demographic variables of unvaccinated and vaccinated patients with severe COVID-19 during the Delta and Omicron stages.

Variable	Delta stage			Omicron stage		
	Unvaccinated (*n* = 131)	Vaccinated (*n* = 74)	*p*-value	Unvaccinated (*n* = 41)	Vaccinated (*n* = 77)	*p*-value
Age, years	52 [42–67]	64 [55–73]	**<0.001**	65 (48–72)	61 (44–72)	0.554
Sex, males *n* (%)	80 (61.07)	47 (63.51)	0.766	25 (60.97)	39 (50.65)	0.334
T2DM, *n* (%)	39 (29.77)	34 (45.94)	**0.023**	11 (26.83)	26 (33.77)	0.534
Obesity, *n* (%)	56 (42.75)	39 (52.70)	0.191	10 (24.39)	33 (42.86)	0.069
COPD, *n* (%)	6 (4.58)	7 (9.46)	0.232	4 (9.76)	5 (6.49)	0.717
HD, *n* (%)	1 (0.76)	2 (2.70)	0.296	5 (12.19)	14 (18.18)	0.445
SAH, *n* (%)	40 (30.53)	37 (50.00)	**0.007**	15 (36.58)	32 (41.56)	0.694
Hosp_stay, days	28 [17–50]	30 [20–47]	0.529	4 (2–8)	4 (3–7)	0.492
Mortality, *n* (%)	31 (23.66)	26 (35.13)	0.758	21 (51.22)	28 (36.36)	0.169
IMV, *n* (%)	109 (83.21)	61 (82.43)	1.000	8 (19.51)	21 (27.27)	0.380
Leukocyte (cell/μl)	9,460 [6,845–9,784]	10,370 [6,728–14,380]	0.134	10,930 (7,950–12,820)	11,040 (7,820–15,800)	0.645
Neutrophil (cell/μl)	7,400 [5,250–10,300]	8,750 [5,300–11,775]	0.080	8,700 (6,390–12,260)	8,930 (5,580–13,400)	0.888
Lymphocyte (cell/μl)	700 [400–1,100]	700 [400–1,100]	0.798	730 (500–1,030)	930 (570–1,470)	0.052
Eosinophils (cell/μl)	0 [0–100]	0 [0–100]	0.827	10 (0–70)	20 (0–120)	0.217
NLR	10.20 [5.85–18.00]	10.15 [6.43–20.88]	0.375	11.62 (7.57–23.58)	11.00 (5.65–19.77)	0.251
Platelets (cell/μl)	272,800 [210,400–362,600]	278,050 [205,000–356,375]	0.987	204,000 (136,000–354,000)	264,000 (188,000–336,000)	0.292
CRP (mg/L)	53.80 [16.11–116.70]	27.45 [8.07–112.55]	0.110	108.2 (60.1–230.6)	108.2 (44.9–237.5)	0.909
LCR	37.09 [8.93–63.90]	34.43 [7.81–96.53]	0.750	5.50 (3.00–14.20)	8.05 (3.21–27.49)	0.312
D-dimer (ng/ml)	1,225 [455–3,000]	810 [430–1,650]	0.345	2,158 (1,078–5,744)	1,911 (1,033–2,705)	0.138

Quantitative data are presented as median (interquartile range Q1–Q3), and qualitative data as absolute numbers (frequency in percentage).

The comparisons were performed using the chi-square and Mann–Whitney U-tests. *p*-values <0.05 are indicated in bold style.

CRD, chronic respiratory diseases; CRP, C-reactive protein; HD, heart diseases; Hosp_stay, days of hospital stay; IMV, invasive mechanical ventilation; LCR, lymphocyte-to-C-reactive protein ratio; NLR, neutrophil-to-lymphocyte ratio; SAH, systemic arterial hypertension; T2DM, type 2 diabetes mellitus.

We also sought to evaluate the effects of anti-SARS-CoV-2 vaccination on clinical and demographic variables of patients hospitalized with COVID-19 during the Omicron stage. At this point, 65.25% (*n* = 77) of the studied patients had received at least one dose of the available COVID-19 vaccine. From this, 11.69% received one dose, 62.3% received two doses, 20.78% received three doses, and 5.19% received four doses.

At the Omicron stage, unvaccinated patients were older and commonly males (60.97%). In general, the frequency of comorbidities was higher among vaccinated patients than among unvaccinated patients. The obesity rate was higher in the vaccinated group (42.86 vs. 24.39%), although this difference was not statistically significant (Table 5).

Regarding patient outcomes with COVID-19, the hospital stay was similar between the two comparison groups, and no statistically significant differences were observed in the percentage of defunctions or IMV requirement. Likewise, cell counts at hospital admission were similar across all cases; only the lymphocyte count was slightly higher in the vaccinated group than in the unvaccinated. The D-dimer levels, which were relevant during the Omicron stage (Table 4), were also similar between groups, although levels were higher in unvaccinated patients than in vaccinated patients.

## 4 Discussion

During the COVID-19 pandemic, there was a relevant heterogeneity in the clinical and demographic variables, as well as in the clinical outcome, which was greatly influenced by the available knowledge about the disease, the evolution of SARS-CoV-2 variants, the vaccination status, and the individual political and social economy of care in the different countries (8, 26, 27). The analysis of retrospective data contributes to understanding the virus's pathophysiology and identifies relevant insights for future health emergencies of viral etiology. Herein, we reported a comparative analysis of clinical and demographic variables across the different COVID-19 waves in Mexico, as well as during the period when some SARS-CoV-2 VOCs were relevant, and the vaccination status during the Omicron stage.

We also reported differences in the clinical and demographic characteristics of patients from two high-specialty centers in Yucatán and Mexico City. Yucatán is characterized by a high prevalence and mortality rate for T2DM (28), and it was also previously identified as a risk factor for COVID-19 death (29). Nevertheless, the frequency of this disease, as well as the frequencies of obesity and systemic arterial hypertension, was no different between the patients from HRAEPY and the INER. Meanwhile, it was notably the high rate of heart diseases among the patients from Yucatan, which could be a regional health problem, and that was influencing COVID-19 mortality, mainly during the Omicron stage. This highlights differences in socioeconomic and cultural conditions across populations within the same country, while also highlighting regional challenges faced during emergent health problems like the COVID-19 pandemic.

At the start of the pandemic, older age was reported as a risk factor for severe COVID-19. Adults over 65 years of age represented 80% of hospitalizations and had an exponentially increased risk of mortality in comparison to younger subjects (30). Several age-related complications appeared to influence this vulnerability, such as immunity decline, heightened baseline inflammation, comorbidities like cardiovascular disease and T2DM, among others (31). We did not observe differences in age among the groups of SARS-CoV-2 variants, but patients hospitalized with severe COVID-19 in the studied hospitals were the youngest in the grouped-wave study (Table 3). The median age was 54 years in the 1st wave; however, obesity had the highest prevalence in this period, which could influence the increased risk of severity in these non-older patients. This agreed with a meta-analysis performed in late 2020, which reported that obesity was associated with a higher risk of severe disease, including hospitalization, need for intensive care unit admission, invasive mechanical ventilation requirement, and mortality (32). Likewise, the highest frequency of men hospitalized due to COVID-19 was observed during the 1st pandemic wave, in which only 30% of the studied patients were women. The male sex was also an early risk factor described during the pandemic, with a relative risk of 1.29 for disease severity and 1.36 for mortality (33).

The prevalence of comorbidities also rose throughout the pandemic. This was especially evident for heart diseases, which became more common among patients hospitalized near the end of the pandemic, infected with an Omicron variant, compared to the first three waves and initial VOCs. This aligns with a previous report in Singapore, where patients with heart failure and ischemic heart disease faced a higher risk of severe COVID-19 and hospitalization compared to matched controls (34). Even a recent meta-analysis including 68 studies found an elevation of relative risk of death, hospitalization, and the combined outcome in patients with cerebrovascular diseases, COPD, T2DM, respiratory diseases, heart disease, and heart failure (35).

In agreement, D-dimer was also a prominent feature of the Omicron stage and the late waves of the COVID-19 pandemic, as we observed it increased compared to other VOCs and COVID-19 waves. D-dimer was reported as a biomarker for mortality risk in patients with SARS-CoV-2 Omicron variant infection complicated with cardiovascular diseases (36); and it was identified as a clinical marker of intubation and mortality since early studies (37). Even during the Omicron stage, unvaccinated patients had the highest D-dimer levels among the analyzed groups (Table 4), reflecting ongoing coagulation and fibrinolysis processes and hemostatic abnormalities such as intravascular thrombosis (38).

The white blood cell analysis showed lymphopenia and neutrophilia during different waves and VOCs. This agreed with an early meta-analysis reporting the relation of lymphopenia and neutrophilia at hospital admission with poor outcomes in patients with COVID-19 (39). Nevertheless, both abnormalities can also be attributed to other common processes during COVID-19, such as corticosteroid use, coexisting bacterial infection, and hyperinflammation (40). Moreover, the lymphopenia associated with SARS-CoV-2 infection has been explained by several mechanisms, including the cytokine storm, which suppresses T-cell proliferation and triggers apoptosis, as well as a potential role for CD147 in the pathogenesis of COVID-19 (41).

On the other hand, inflammation was a core problem in severe COVID-19. In some cases, the host response showed extrapulmonary systemic hyperinflammation, characterized by elevations of interleukins (i.e., IL-2, IL-6, IL-7), tumor necrosis factor-α, CRP, ferritin, D-dimer, and high-sensitivity cardiac troponin I (42). This hyperinflammation played a critical role in the severity and clinical outcome of the disease, and it has been related to the development of thrombotic complications, such as pulmonary embolism, deep vein thrombosis, and strokes (43). In our study, systemic inflammation was consistently observed, with elevated CRP and LCR; the highest CRP values occurred during the 1st wave, and the lowest were associated with the Delta variant. Nevertheless, it is worth noting that all patients included were hospitalized with varying degrees of COVID-19 severity, and the inflammation status was expected.

We also observed a higher mortality rate in the last waves of the COVID-19 pandemic and during the Omicron stage, compared to the early stages of the emergency. Controversial findings have been reported in the literature in this regard. Although COVID-19 severity was less severe with the spread of the Omicron variant (44, 45), the number of in-hospital deaths did not decrease as expected (46). A recent large-scale real-world data analysis found that deaths increased during the Omicron-pre-dominant wave, with age as the explanatory factor (47). In agreement, we observed an increase in age across the 4th to 6th waves, as well as in the Omicron variant, and a high prevalence of heart disease as a mortality risk factor, as previously discussed.

Finally, several reports have shown decreases in mortality rates and COVID-19 severity after anti-SARS-CoV-2 vaccination with both complete and incomplete schemes (48–51). During the Delta stage, most patients had not received a COVID-19 vaccine, and the majority of patients in the vaccinated group had received only one dose. In comparing the groups, we observed only prioritization of the vulnerable population (older adults with comorbidities) for vaccine administration, with no effect on mortality or severity. In the Omicron stage, a higher proportion of patients had received two doses, but we did not observe differences in clinical or demographic variables by vaccination status; however, the mortality rate was lower in the vaccinated group than in the unvaccinated group. This is consistent with previous studies reporting decreased immune responses after anti-SARS-CoV-2 vaccination in older adults and those with comorbidities (52, 53), both of which are prevalent in our population. Further studies are warranted to evaluate the effects of clinical and demographic characteristics on response to COVID-19 vaccines.

Our study is not exempt from limitations. This study is primarily limited by its retrospective design and the heterogeneity in the number of subjects across comparison groups. We could not include some relevant data (e.g., the history of prior infections with SARS-CoV-2 variants, the number of days since symptom onset to the date of laboratory testing, social factors influencing the selection process, etc.) due to the study's retrospective design. We also could not specify the SARS-CoV-2 variant infecting the studied patients because these analyses were not performed for all cases, and the results were not included in the electronic medical records. Likewise, we lack information on pharmacological management; although the use of antivirals was not frequent in our country, the use of antibiotics, corticosteroids, and anticoagulants could help explain the differences in clinical outcomes. However, we observed differences in clinical and demographic variables across pandemic waves and SARS-CoV-2 variants that could help to explain the variability in clinical outcomes throughout the COVID-19 pandemic.

## Data Availability

The raw data supporting the conclusions of this article will be made available by the authors, without undue reservation.
