# Supplementary material for: An outlook on clinical and demographic variables in patients with severe COVID-19 across different waves in Mexico and SARS-CoV-2 variants: data from two high-specialty centers
Source: Front Public Health. 2026 Apr 23;14:1794262. doi: 10.3389/fpubh.2026.1794262 (PMC13149431; doi:10.3389/fpubh.2026.1794262)
Supplement: Supplementary file 1 [file Supplementary_file_1.docx]

**Supplementary material**


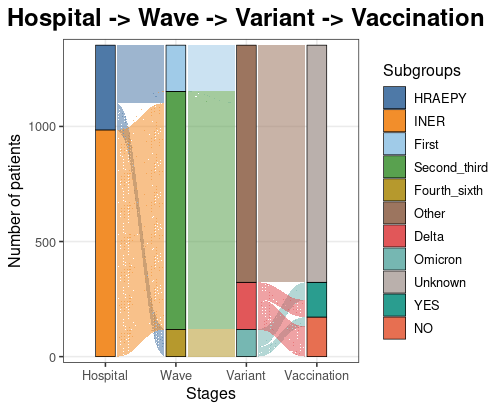


**Supplementary Figure 1. Sankey plot showing the flow of patients from each high-specialty center to the different grouped waves, SARS-CoV-2 variants, and the vaccination status.** Figure created in RStudio.
